# Quantitative Nature of Social Vulnerability and Autism: An Important Paradigm Shift in the DSM-5 for Autism Spectrum Disorder

**DOI:** 10.1155/2013/201719

**Published:** 2013-05-08

**Authors:** Shinji Ijichi, Naomi Ijichi, Yukina Ijichi, Kazumi Hirotaki, Hisami Sameshima, Yoichi Kawaike, Hirofumi Morioka

**Affiliations:** ^1^Health Service Center, Kagoshima University, 1-21-24 Korimoto, Kagoshima 890-8580, Japan; ^2^Institute for Externalization of Gifts and Talents (EGT), 7421-1 Shimofukumoto-cho, Kagoshima 891-0144, Japan

## Abstract

In the Diagnostic and Statistical Manual of Mental Disorders, 4th edition (DSM-IV), autistic characteristics in social interaction and communication are described as qualitative impairments. However, the difference between autistics and nonautistics in the draft of the 5th edition (DSM-5 draft) is quantitative rather than qualitative. The word “qualitative” is deleted in the draft text, and it is specified that the relation between social demands and individual limited capacities is critical for symptom manifestation (criterion C). Because the proposed levels of support requirement in the draft are mere observable outcomes of social vulnerability, the boundary between level 1 and nonautistic condition is determined by the relation between social demands and individual capacities. In addition to the introduction of the single category (autism spectrum disorder (ASD)) to cover the entire case spectrum, the DSM-5 draft is clearly based on a conviction that ASD is indistinguishable from the normal behavioral range. This concise review provides an explanation for this implicit paradigm shift from qualitative to quantitative. Importantly, the conditional role of social demands for symptom manifestation in the draft can be plausibly interpreted using a unique liability-probability model.

## 1. Introduction

The development process of the fifth edition of the Diagnostic and Statistical Manual of Mental Disorders (DSM-5) is nearing its conclusion, and it is already scheduled for release in May 2013 (http://www.dsm5.org/). Although there has been a lot of debate on the new draft criteria for autism spectrum disorder (ASD) [1–3], the official commentaries from the DSM-5 workgroup on neurodevelopmental disorders suggest minor additional revisions in the upcoming final version [4]. Because the proposals in the draft are supported by more than a decade of scientific evidences after the launch of DSM-IV, the implications in the draft proposals are significant, and it is quite meaningful to designate the implicit differences between the draft and the former version.

In DSM-IV, autistic characteristics in social interaction and communication are described as qualitative impairments. However, the difference between autistics and nonautistics in the DSM-5 draft is quantitative rather than qualitative. The word “qualitative” is deleted in the draft text, and it is suggested that the diagnosis of ASD may not be confirmed until social demands exceed limited capacities as documented in the criterion C. The proposed levels of support requirement in the draft are mere observable outcomes of social vulnerability. Therefore, the boundary between level 1 ASD and non-autistic condition is determined by the relation between social demands and individual capacities. The DSM-5 draft is clearly based on a conviction that ASD is indistinguishable from the normal behavioral range. This important paradigm shift to a quantitative construction on autism is implicitly introduced in the draft. Here we challenge to illustrate the backgrounds of this paradigm alteration and introduce a unique liability-probability model to explain the entire structure of autism.

## 2. Autism and Biological Markers

Autism is a developmental lifelong condition of the human brain, and a behavioral characterization as a spectrum is the best way to define this complex trait [5]. The predominant presence of autistic cases without comorbidity [6] clearly means that the biological effects associated with the known concomitant medical conditions (cytogenic abnormalities, fragile X syndrome, tuberous sclerosis, congenital infection, maternal thalidomide use, epilepsy, etc.) cannot be the common prerequisite for autism at least in the majority of the cases. The involvement of genetic factors is evident from the results of twin study, and many gene variants which seem to affect brain development and synaptic functions have been reported in association with ASD [7]. However, the reported gene variants are, at present, nothing but one of the concomitants in a small percentage of the cases [8]. There is as yet no qualitative biological marker which is cosegregated with ASD, and even the molecular deviations associated with the reported gene variants have not been confirmed [8]. Because the apparent involvement of genetic factors or high heritability does not vindicate the condition as a diagnostic category [9], the evolutionary survived trait may be the dimensional diversity itself [8].

## 3. Two-Dimensional Properties of Autism in the Draft of DSM-5

While the draft of DSM-5 is at the final revision stage, the core amendments associated with ASD seem to be reserved for the May 2013 release [4]. The core proposals in the draft include introduction of the single category to cover the entire case spectrum, unification of the social and communication criteria, creation of severity criteria, and development of a new related category (social communication disorder). Although the introduction of the case spectrum named ASD attracts a vast researcher's attention [1–3], the other important conceptual alteration associated with the whole dimension of social vulnerability is implicitly introduced to DSM-5, as summarized the following (Section 3.2).

### 3.1. The ASD Case Spectrum Which Incorporates DSM-IV Separate Subtypes

The potential benefits for research and clinical practice of incorporating a dimensional component into the existing categorical classification system in the DSM have been underscored in the review and planning process for DSM-5 [10]. The presence of the continuity between typical autistic cases and the so-called other subtypes had consistently been recognized [1, 5, 11]. However, in order to increase the apparent reliability across diagnosticians, related diagnoses including Asperger's disorder, childhood disintegrative disorder, and pervasive developmental disorder not otherwise specified had been introduced in DSM-IV (1994) as distinct categories. This position, which has sometimes caused a misunderstanding that there are clear clinical distinctions among diagnostic subtypes of ASD [12], has completely been changed in the draft of DSM-5. The proposal by the neurodevelopmental work group recommends to employ a dimensional umbrella, ASD, which would incorporate previously separate diagnoses, including autistic disorder, Asperger's disorder, childhood disintegrative disorder, and pervasive developmental disorder not otherwise specified. The move from subgrouping to the unifying description is supported by some recent studies [13–16]. In addition, in our opinion, hyperactive children with a diagnosis of attention-deficit/hyperactive disorder (AD/HD) have also difficulty in social communication and obsessive traits and should be involved in this ASD case spectrum [17].

### 3.2. The Whole Dimension

Excellent authorities appreciated the smooth behavioral continuum between individuals with ASD and the non-autistic majority [5, 18], but autistic characteristics have sometimes been represented as qualitative impairments [19]. DSM-IV specified that the behavioral deficits in social interactions and communication must be qualitatively out of proportion to the individual's cognitive level, without defining the difference in quantitative terms [20]. Although it was repeatedly emphasized that the supposed qualitative diagnostic segregation can be judged by an experienced clinician [5, 21], there is no evidence that any aspect of the autistic phenotype is qualitatively distinct from normal development. The term “qualitative” was used in the categorical approach to illustrate the range of impairments [19] on the assumption that the autistic behavioral range is qualitatively distinguishable from the normal range. Many population studies challenged this assumption and revealed that ASD is best characterized as an extreme of a bell-shaped behavioral dimension that distribute quantitatively from normal development to autistic development [22–31]. In the DSM-5 draft for ASD, there is no description of “qualitative,” and it is demonstrated that a balance between social demands and individual limited capacities is critical for the diagnosis. If social demands do not exceed limited capacities in an individual, autistic symptoms may not become fully manifest (Criterion C), and if educational or social support is not required because of the fewer social demands, the condition cannot fulfill the criteria for the lowest severity level (Level 1). In contrast to DSM-IV, the DSM-5 draft is clearly based on a conviction that ASD is indistinguishable from the normal behavioral range.

### 3.3. Subdimensions in the Whole Dimension

Proposed changes in the DSM-5 draft include reducing the domains from three to two by merging the social interaction and communication domains into a single domain (social communication domain). Regarding the three domains of DSM-IV, population-based quantitative studies have provided important information which suggests that the phenotypic components of the triad may arise from different genetic or environmental clusters [25, 26, 32]. This complex structure was illustrated as a “family of dimensional phenotypes” that includes symptoms and level of functioning [33]. Because the modest correlations between each subdimension [25, 26, 32] may make it troublesome to illustrate each implication in the whole dimension, a putative parameter which can indicate the degree of net autistic characteristics in each individual is postulated as the whole dimension in this paper for further consideration.

## 4. Population Settings and Clinical Settings

In spite of the converging evidence that ASD is best characterized as an extreme of a bell-shaped behavioral dimension that distribute quantitatively from normal development to autistic development [22–31], ASD can still be documented as a categorical entity in clinically ascertained samples [34]. This fact in clinical settings may be the underpinning for the qualitative or categorical definitions in DSM-IV. In order to illustrate the backgrounds of the important paradigm alteration in DSM for autism from qualitative to quantitative, samples of research data should be divided according to the sampling fields, population settings or clinical settings. Population settings provide the field of population studies, population-based twin studies, screening studies, and community sample studies. Clinical settings involve the field of clinically ascertained studies, proband-based studies, outpatient studies, and institutionalized sample studies.

## 5. Backgrounds of the Paradigm Shift from Qualitative to Quantitative

**Figure 1:**
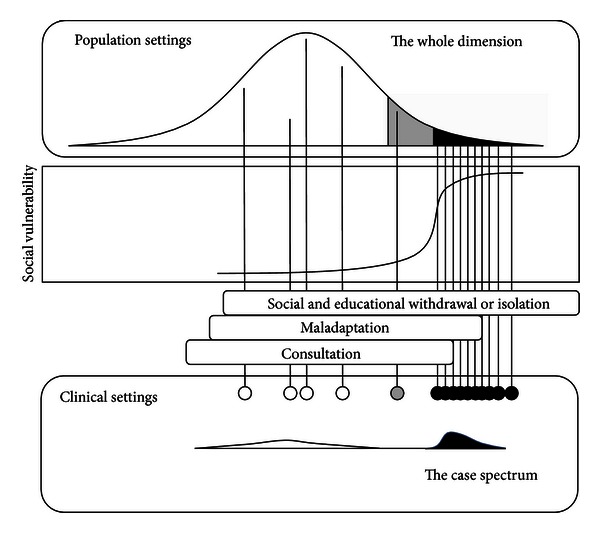
Two-dimensional properties of autism in different settings. The first one (the case spectrum) in clinical settings is designated as a small skewed distribution (black) at the bottom right of the figure. The other (the whole dimension) in population settings is located at the top of the figure, which has a sigmoid relationship with social vulnerability and involves both nonautistics and autistics as described in text. Under this liability-probability model, the vulnerability is low but not zero in the majority (white part), modest in the border zone (grey), and very high but not the maximum in the extreme tail (black).

In the traditional liability-threshold model, susceptibility to a condition, where there are two apparent phenotypic classes, affected or not affected, is recognized as an underlying dimensional continuity (liability) with a threshold, which imposes a discontinuity on the visible expression [35]. Here, the putative parameter which can indicate the degree of net autistic characteristics in each individual is introduced as described previously, and a sigmoid relationship is assigned between the autistic continuity and the social vulnerability (Figure 1). To interpret the DSM-5 criterion, “symptoms may not become fully manifest until social demands exceed limited capacities,” this vulnerability value should be mere a probability, and the actual maximum value is less than 1.0 in this sigmoid model. Therefore, there is no absolute threshold in this liability-probability model.

The two distributional dimensions are illustrated in the Figure 1. As reviewed previously, the first one (the case spectrum) is well considered in the DSM-5 draft as a unifying category, ASD. The other (the whole dimension) has a sigmoid relationship with the social vulnerability. ASD merges into what can be called eccentric normality (the border zone) and there is no clear cutoff point as described by Wing [5]. Importantly, even in the autistic extreme tail, only if social demands exceed their limited capacities, the autistic individuals can be resigned to the susceptibility resulting in maladaptation, socioeducational withdrawal, or isolation, and the autistic symptoms can become fully manifest, as described in the DSM-5 draft. Because ASD cases can be easily distinguished from non-ASD cases in clinical settings, both diagnostic accuracy and interrater reliability are high enough for the expert diagnosticians to enjoy the applause as “true gold standard” (diagnosis) providers [4]. In population settings, however, especially for individuals in whom social demands do not exceed their limited capacities, both autistic characteristics and the individual social vulnerability cannot be the absolute determinant to accurately expect coming limitation of everyday functioning.

## 6. Discussions

The significance of a balanced sampling in both settings, population and clinical settings, was already suggested by several research authors [3, 34]. The referral bias can influence sensitivity and specificity in studies to evaluate criteria [3]. Especially in taxometric studies, which are usually used to distinguish a categorical model from a dimensional model, population-based samples may be critical for a comprehensive understanding [34]. DSM-5 may have a broader perspective to view the entire situation of autism researches than former criteria, and in the draft the case spectrum in clinical settings and the conceptual alteration concerning social vulnerability are both introduced as described previously. This concise review provides an explanation for the conflict between the qualitative framework [34] and the quantitative framework [22–31]. Importantly, the conditional role of social demands for symptom manifestation in the draft can be plausibly interpreted using a unique liability-probability model.

In population settings, none of the autism screening tests has been shown to be able to fulfill the properties of accuracy [36]. The difficulty in the implementation of a routine population-based screening program for autism can be explained from our perspective. As described in the DSM-5 draft, even in the autistic extreme tail, only if social demands exceed their limited capacities, the autistic individuals can be resigned to the susceptibility resulting in maladaptation, socioeducational withdrawal, or isolation, and the autistic symptoms can become fully manifest. For individuals in whom social demands do not exceed their limited capacities, both autistic characteristics and the individual social vulnerability cannot be the absolute determinant to accurately expect coming limitation of everyday functioning. Therefore, the occurrence of misdiagnosis and overdiagnosis may be inevitable in screening programs using available tools in population settings [36].

In proband-based family studies, the probands are cases in clinical settings, but the nonprobands (siblings, cotwins, etc.) are in population settings with a sampling bias. The low compatibility of recent results with previous data regarding sibling recurrence risk and twin concordance rates may be partly attributable to the poor interrater agreement in the diagnostic decisions for nonprobands [37, 38].

## 7. Conclusions

In 1999, the research on autism was metaphorically depicted as a situation in which “myopic investigators are still patting the elephant” [20]. Can we say now that the confused state is merely historical [33, 39]? DSM-5 for ASD may at least have a broader perspective to view the entire structure than former criteria, and in the draft the case spectrum in clinical settings and the conceptual alteration concerning social vulnerability are both introduced. We hope that these proposals warrant further constructive considerations from broader viewpoints after the launch of DSM-5.

## References

[1] Wing L, Gould J, Gillberg C (2011). Autism spectrum disorders in the DSM-V: better or worse than the DSM-IV?. *Research in Developmental Disabilities*.

[2] Taheri A, Perry A (2012). Exploring the proposed DSM-5 criteria in a clinical sample. *Journal of Autism and Developmental Disorders*.

[3] McPartland JC, Reichow B, Volkmar FR (2012). Sensitivity and specificity of proposed DSM-5 diagnostic criteria for autism spectrum disorder. *Journal of the American Academy of Child & Adolescent Psychiatry*.

[4] Swedo SE, Baird G, Cook EH (2012). Commentary from the DSM-5 workgroup on neurodevelopmental disorders. *Journal of the American Academy of Child & Adolescent Psychiatry*.

[5] Wing L (1997). The autistic spectrum. *The Lancet*.

[6] Freitag CM (2007). The genetics of autistic disorders and its clinical relevance: a review of the literature. *Molecular Psychiatry*.

[7] Betancur C (2011). Etiological heterogeneity in autism spectrum disorders: more than 100 genetic and genomic disorders and still counting. *Brain Research*.

[8] Ijichi S, Ijichi N, Ijichi Y, Sameshima H, Morioka H, Deutsch SI, Urbano MR (2011). The genetic basis of phenotypic diversity: autism as an extreme tail of a complex dimensional trait. *Autism Spectrum Disorders: The Role of Genetics in Diagnosis and Treatment*.

[9] Keller MC, Miller G (2006). Resolving the paradox of common, harmful, heritable mental disorders: which evolutionary genetic models work best?. *Behavioral and Brain Sciences*.

[10] Regier DA (2007). Dimensional approaches to psychiatric classification: refining the research agenda for DSM-V: an introduction. *International Journal of Methods in Psychiatric Research*.

[11] Waterhouse L, Wing L, Spitzer R, Siegel B (1992). Pervasive developmental disorders: from DSM-III to DSM-III-R. *Journal of Autism and Developmental Disorders*.

[12] Simpson D (2003). Autism spectrum disorder is not as certain as implied. *British Medical Journal*.

[13] Kamp-Becker I, Smidt J, Ghahreman M, Heinzel-Gutenbrunner M, Becker K, Remschmidt H (2010). Categorical and dimensional structure of autism spectrum disorders: the nosologic validity of asperger syndrome. *Journal of Autism and Developmental Disorders*.

[14] Lord C, Petkova E, Hus V (2012). A multisite study of the clinical diagnosis of different autism spectrum disorders. *Archives of General Psychiatry*.

[15] Frazier TW, Youngstrom EA, Speer L (2012). Validation of proposed DSM-5 criteria for autism spectrum disorder. *Journal of the American Academy of Child & Adolescent Psychiatry*.

[16] Huerta M, Bishop SL, Duncan A, Hus V, Lord C (2012). Application of DSM-5 criteria for autism spectrum disorder to three samples of children with DSM-IV diagnoses of pervasive developmental disorders. *American Journal of Psychiatry*.

[17] Ijichi S, Ijichi N (2007). Computerized lifelong mentoring support using robot for autistic individuals. *Medical Hypotheses*.

[18] Rapin I (1997). Autims (author reply). *The New England Journal of Medicine*.

[19] Filipek PA, Accardo PJ, Baranek GT (1999). The screening and diagnosis of autistic spectrum disorders. *Journal of Autism and Developmental Disorders*.

[20] Rapin I (1999). Autism in search of a home in the brain. *Neurology*.

[21] Filipek PA, Accardo PJ, Ashwal S (2000). Practice parameter: screening and diagnosis of autism. Report of the quality standards subcommittee of the American Academy of Neurology and the Child Neurology Society. *Neurology*.

[22] Constantino JN, Todd RD (2000). Genetic structure of reciprocal social behavior. *American Journal of Psychiatry*.

[23] Skuse DH, Mandy WPL, Scourfield J (2005). Measuring autistic traits: heritability, reliability and validity of the Social and Communication Disorders Checklist. *British Journal of Psychiatry*.

[24] Ronald A, Happé F, Plomin R (2005). The genetic relationship between individual differences in social and nonsocial behaviours characteristic of autism. *Developmental Science*.

[25] Ronald A, Happé F, Bolton P (2006). Genetic heterogeneity between the three components of the autism spectrum: a twin study. *Journal of the American Academy of Child and Adolescent Psychiatry*.

[26] Ronald A, Happé F, Price TS, Baron-Cohen S, Plomin R (2006). Phenotypic and genetic overlap between autistic traits at the extremes of the general population. *Journal of the American Academy of Child and Adolescent Psychiatry*.

[27] Posserud MB, Lundervold AJ, Gillberg C (2006). Autistic features in a total population of 7-9-year-old children assessed by the ASSQ (Autism Spectrum Screening Questionnaire). *Journal of Child Psychology and Psychiatry and Allied Disciplines*.

[28] Hoekstra RA, Bartels M, Verweij CJH, Boomsma DI (2007). Heritability of autistic traits in the general population. *Archives of Pediatrics and Adolescent Medicine*.

[29] Constantino JN (2011). The quantitative nature of autistic social impairment. *Pediatric Research*.

[30] Robinson EB, Koenen KC, McCormick MC (2011). Evidence that autistic traits show the same etiology in the general population and at the quantitative extremes (5%, 2.5%, and 1%). *Archives of General Psychiatry*.

[31] Lundstöm S, Chang Z, Råstam M (2012). Autism spectrum disorders and autistic like traits: similar etiology in the extreme end and the normal variation. *Archives of General Psychiatry*.

[32] Robinson EB, Koenen KC, McCormick MC (2012). A multivariate twin study of autistic traits in 12-year-olds: testing the fractionable autism triad hypothesis. *Behavior Genetics*.

[33] Szatmari P (2011). New recommendations on autism spectrum disorder. *British Medical Journal*.

[34] Frazier TW, Youngstrom EA, Sinclair L (2010). Autism spectrum disorders as a qualitatively distinct category from typical behavior in a large, clinically ascertained sample. *Assessment*.

[35] Falconer DS, Mackay TFC, Falconer DS, Mackay TFC (1996). Threshold characters. *Introduction to Quantitative Genetics*.

[36] Al-Qabandi M, Gorter JW, Rosenbaum P (2011). Early autism detection: are we ready for routine screening?. *Pediatrics*.

[37] Hallmayer J, Cleveland S, Torres A (2011). Genetic heritability and shared environmental factors among twin pairs with autism. *Archives of General Psychiatry*.

[38] Ozonoff S, Young GS, Carter A (2011). Recurrence risk for autism spectrum disorders: a baby siblings research consortium study. *Pediatrics*.

[39] Voelker R (2011). Autism screening strikes emotional chord. *Journal of the American Medical Association*.

